# A Visit with Dr. Louis Woolf, Recognizing His 100th Birthday and His Contributions to the Diagnosis and Treatment of Phenylketonuria

**DOI:** 10.3390/ijns6020045

**Published:** 2020-05-30

**Authors:** R. Rodney Howell, Graham Sinclair

**Affiliations:** 1 Department of Pediatrics, Miller School of Medicine, University of Miami, Miami, FL 33136, USA; 2 Department of Pathology and Laboratory Medicine, University of British Columbia, Vancouver, BC V6H 3N1, Canada; gsinclair@cw.bc.ca

One of the most dramatic discoveries in metabolic disease research was that of Ashbørn Følling, who in 1934, published his research outlining unusual biochemical findings in a set of siblings with severe developmental delay [1]. In these Norwegian children, and in others that he soon identified, there was a striking accumulation of the essential amino acid phenylalanine. He named the condition phenylpyruvic oligophrenia. Since the accumulating amino acid was essential, at the time it was considered that the dietary limitation of this amino acid might be beneficial to those affected. Over the ensuing years, a variety of dietary manipulations were tried, as outlined in another article in this journal on the contributions of Dr. Louis I. Woolf. The critical role that Woolf played in the development of the experimental diet, first used by Horst Bickel and his colleagues, is recognized in a classic note of appreciation by Bickel, published in 1953, and shown here in Figure 1 [1]. It is also important to note that, in this same recognition, Allen and Hanburys, Ltd. is thanked for providing phenylalanine-free casein hydrolysate. Dr. Woolf was employed as a chemist for this group, where he developed these materials.

At a meeting of the Council of the International Society for Neonatal Screening, it was decided that this organization would present a plaque to Dr. Louis I. Woolf, recognizing his seminal contributions to the treatment of phenylketonuria, on his 100th birthday. The discovery of phenylketonuria and an effective treatment, which required that it be started in the newborn period, is, of course, the basis for the development of population-based, neonatal screening.

Prior to his retirement, Dr. Woolf had assumed a role at the University of British Columbia in Vancouver, Canada, and he has continued to live in the beautiful city. I (R. Howell) had previously presented Dr. Woolf with an award on his 90th birthday, honoring his contributions, from The American College of Medical Genetics in the United States. Since I had met Dr. Woolf earlier, and in 2019 was the president of ISNS, it was my great pleasure to travel to Vancouver and present him with an appropriate plaque, as shown in Figure 2. I was joined by another ISNS member, Dr. Graham Sinclair, Clinical Professor of Pathology and Laboratory Medicine and Director of the Newborn Screening Laboratory at BC Children’s Hospital and the University of British Columbia, as shown in Figure 3.

On a beautiful late summer day in 2019 in Vancouver, we had the privilege of meeting with Dr. Woolf. He resides in a garden-like facility, surrounded by spacious grounds, wonderful outdoor views, and a thoughtful and attentive staff. His daughter Leslie, who lives in Vancouver with her husband and can visit readily, was also present, as shown in Figure 4.

It was a wonderful visit, and Dr. Woolf’s memory of the critical events leading up to the effective treatment of PKU was remarkable. Dr. Sinclair carefully recorded the visit and photographs are attached. Dr. Woolf recounted, with great clarity, humor, and compassion, his first experiences with patients with PKU and his early recognition of the potential treatability of this condition. His vision regarding the possibility of using charcoal filtering to deplete phenylalanine from casein hydrolysate was key in the first attempts, by his friend and colleague, Dr. Horst Bickel, to treat PKU with a phenylalanine-restricted diet. Dr. Woolf recalled his continued work to refine this approach and his use of early developmental assessment tools to provide evidence that the earlier the diet was started, the better the outcomes for those affected. This led Dr. Woolf to become a strong advocate for newborn screening and he described his efforts in the establishment of urine phenylalanine screening for newborns in the UK. His contributions to the treatment and screening of infants are many and have had a great impact. We thanked Dr. Woolf on behalf of the ISNS for his seminal contributions, which continue to benefit newborns around the world.

## Figures and Tables

**Figure 1:**
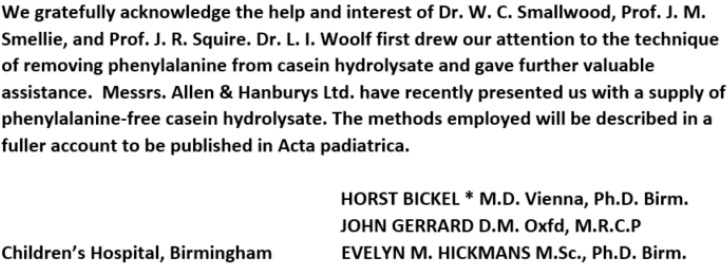
The acknowledgement in an original communication about phenylalanine intake in phenylketonuria [2].

**Figure 2:**
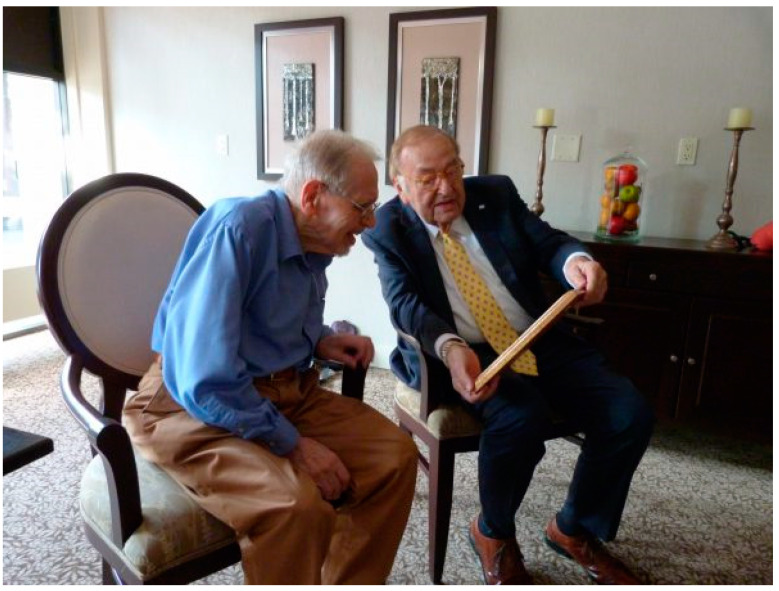
Dr. Howell reviewing the wording with Dr. Woolf of the plaque presented by the ISNS. The wording on the plaque was as follows: Dr. Louis I. Woolf—To recognize and appreciate the vision, expertise and persistence of Dr. Louis I. Woolf for helping; To lay the foundations for the successful dietary treatment of phenylketonuria (PKU). On behalf of the families and individuals all around the world who have benefited, his colleagues and members of the International Society for Newborn Screening would like to recognize and pay tribute to Dr. Woolf for this inspirational achievements which have helped to change the lives of so many in such a profound way and for opening a new path in public health initiating a chapter in health and preventive medicine.

**Figure 3:**
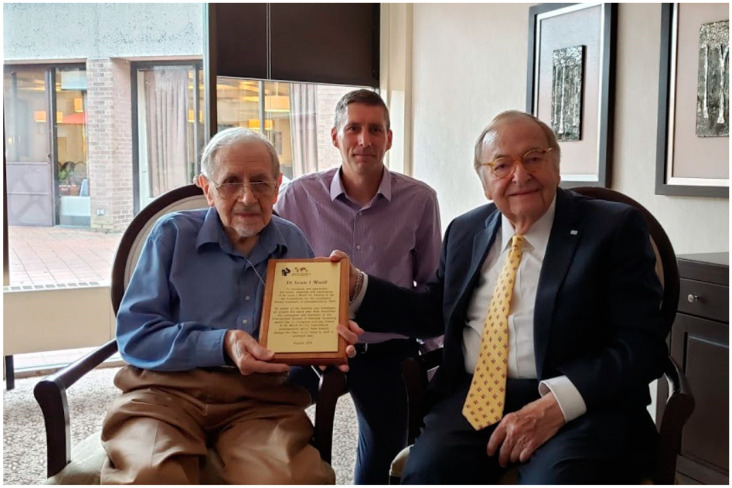
Dr. Graham Sinclair (Center) and Dr. R. Rodney Howell (Right) presenting the ISNS Award to Dr. Woolf (Left).

**Figure 4:**
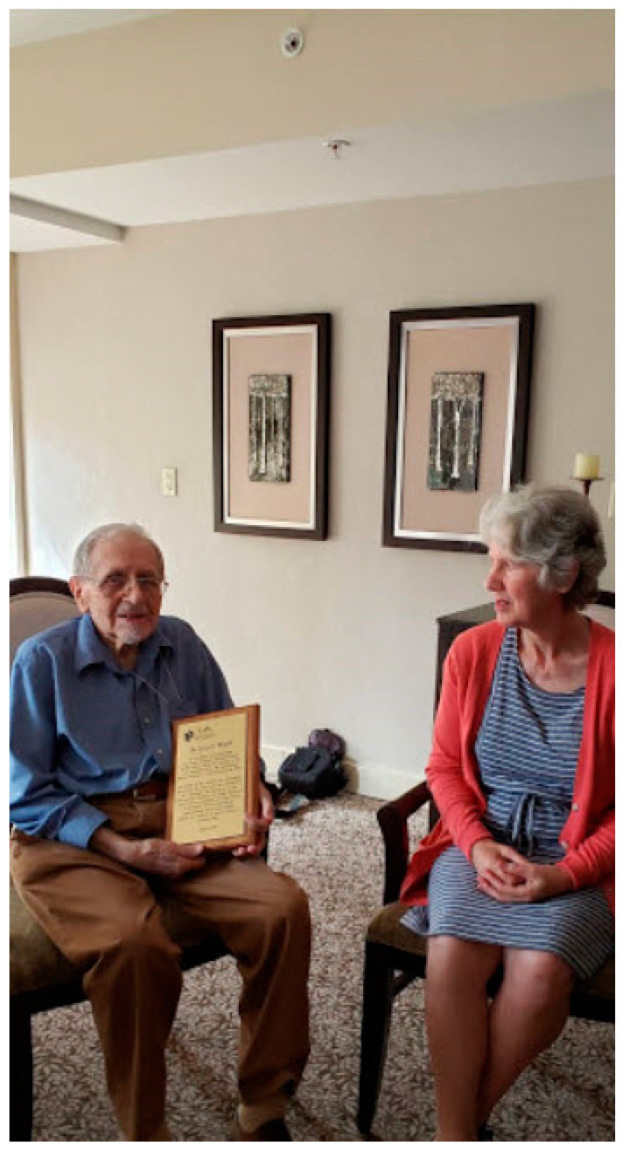
Dr. Woolf displaying his plaque to his daughter, Mrs. Leslie Weston. We had the opportunity to speak directly with Dr. Woolf by telephone about our upcoming visit and we were enormously helped in our arrangements by Dr. Woolf’s daughter, Mrs. Weston.
